# Increased Expression of Phosphorylated Polo-Like Kinase 1 and Histone in Bypass Vein Graft and Coronary Arteries following Angioplasty

**DOI:** 10.1371/journal.pone.0147937

**Published:** 2016-01-28

**Authors:** Swastika Sur, Vicki J. Swier, Mohamed M. Radwan, Devendra K. Agrawal

**Affiliations:** 1 Department of Biomedical Sciences, Creighton University School of Medicine, Omaha, NE, United States of America; 2 Department of Clinical & Translational Science, Creighton University School of Medicine, Omaha, NE, United States of America; University of Central Florida, UNITED STATES

## Abstract

Interventional procedures, including percutaneous transluminal coronary angioplasty (PTCA) and coronary artery bypass surgery (CABG) to re-vascularize occluded coronary arteries, injure the vascular wall and cause endothelial denudation and medial vascular smooth muscle cell (VSMCs) metaplasia. Proliferation of the phenotypically altered SMCs is the key event in the pathogenesis of intimal hyperplasia (IH). Several kinases and phosphatases regulate cell cycle in SMC proliferation. It is our hypothesis that increased expression and activity of polo-like kinase-1 (PLK1) in SMCs, following PTCA and CABG, contributes to greater SMC proliferation in the injured than uninjured blood vessels. Using immunofluorescence (IF), we assessed the expression of PLK1 and phosphorylated-PLK1 (pPLK1) in post-PTCA coronary arteries, and superficial epigastric vein grafts (SEV) and compared it with those in the corresponding uninjured vessels. We also compared the expressions of mitotic marker phospho-histone, synthetic-SMC marker, contractile SMC marker, IFN-γ and phosphorylated STAT-3 in the post-PTCA arteries, SEV-grafts, and the uninjured vessels. Immunostaining demonstrated an increase in the number of cells expressing PLK1 and pPLK1 in the neointima of post PTCA-coronary arteries and SEV-grafts compared to their uninjured counterparts. VSMCs in the neointima showed an increased expression of phospho-histone, synthetic and contractile SMC markers, IFN-γ and phosphorylated STAT-3. However, VSMCs of uninjured coronaries and SEV had no significant expression of the aforementioned proteins. These data suggest that PLK1 might play a critical role in VSMC mitosis in hyperplastic intima of the injured vessels. Thus, novel therapies to inhibit PLK1 could be developed to inhibit the mitogenesis of VSMCs and control neointimal hyperplasia.

## Introduction

Cell division, and the progress of cells through their respective cell cycles; are finely regulated and controlled, at specific junctures, by a complex interplay between various kinases and phosphatases. Understanding the critical role of these kinases and phosphatases, as the major regulators of the cell cycle, marked the start of a new era of increased comprehension of cell cycle progression. Polo like kinase 1 (PLK1), a member of the polo like kinase family, is a serine/threonine kinase that regulates cell cycle progression and mitosis [1]. PLK1 has been reported to mediate multiple mitotic processes including centrosome maturation, assembly of the bipolar spindle, chromosome segregation, activation of the anaphase promoting complex (APC/C), and cytokinesis [2–9]. The expression and activity of PLK 1 is elevated in tissues and cells with high mitotic index, such as cancer cells. Cancers including breast, ovarian, endometrial, prostate, and colorectal are found to have such elevated expression and activity of PLK1 [10–14]. In 2012, Shan and colleagues [15] reported increased expression of PLK1 and its upstream regulator Aurora A kinase in uterine leiomyosarcomas, a neoplastic lesion involving SMCs of the uterine myometrium. Targeting and inhibition of the PLK1 upstream kinase, Aurora A, in the leiomyosarcomas cells decreased the proliferation and induced apoptosis in these malignant cells [15].

Vascular smooth muscle cells (VSMCs) found in the media of coronary arteries play a critical role in the pathogenesis of intimal hyperplasia (IH), which proceeds percutaneous coronary angioplasty (PTCA) and coronary artery bypass surgery (CABG) [16–18]. Angioplasty and bypass grafting are the two major interventional procedures employed to re-vascularize coronary arteries that are occluded, secondary to coronary artery disease (CAD). The aforementioned coronary vascular interventions injure the vascular wall and cause endothelial denudation, and medial VSMCs metaplasia: from contractile/quiescent to a synthetic/proliferative state [19–21]. The phenotypically transformed medial SMCs are the central effectors of IH [19–21]. These highly proliferative and migrating SMCs expresses higher levels of synthetic markers including non-muscle myosin heavy chain B (SMemb), relative to contractile SMCs [22]. Several mitogens such as interferon gamma (IFNᵞ) released at the site of the injury stimulate the medial SMCs to enter G1 phase of cell cycle followed by the subsequent DNA synthesis at the S phase [23, 24]. The expression of PLK1 begins to increase from S/G2 phase and peaks at mitosis in cancerous cells. Phosphorylated-signal transduction and transcription (pStat-3) is implicated as the transcriptional factors of the *PLK1* gene [25–27]. Structurally PLK1 has two domains; one is a C-terminal polo box domain targeting substrate for sub-cellular localization regulating the functional aspect of PLK1 and the other one is the kinase domain that gets activated upon phosphorylation by other kinases. Once activated, PLK1 phosphorylates and further activates a wide range of substrates including Cdc25, Cyclin B1, and CDK1, which promotes mitotic progression [28, 29]. Therefore, PLK1 acts as a hub, where it receives several signals at a specific time and place and in turn reacts by transmitting multiple signals to the effector proteins. Given its well-established functions in regulating cell cycle and promoting proliferation in human cancers, we singled out PLK1 for further study in hyperplastic VSMC.

We focused our initial efforts on examining the role of PLK1 for several reasons: first, PLK1 functions in activating the major kinases, phosphatases and cyclins that promote mitotic entry and progression through the G2-M cell cycle check point, [29] second, PLK1 overexpression has been well documented in cells with high mitotic index including an SMC tumor, leiomyosarcoma [15], third, a number of small ATP-competitive inhibitors such as BI 2536 and BI 6727, highly selective for PLK 1, are currently in different phases of clinical trial against other common human cancers [30, 31]. We have investigated the presence, expression and activity of PLK1 in VSMCs, which may play a critical role in the development of IH after PTCA and CABG in a porcine model. In this investigation, we compared the immunostaining pattern of PLK1, phosphorylated-PLK 1 (pPLK1), SMemb, α-SMA, IFNᵞ, and pStat-3 in a porcine model post PTCA and CABG with their respective uninjured vessels as control. In CABG model of swine, the left and right superficial epigastric veins (SEVs) were chosen to mimic the branches of the main human saphenous vein because their sizes are similar to these branches and can be easily harvested. Since the vascular injury caused by PTCA or CABG produces SMC phenotype switching, and increased proliferation of vascular SMCs leading to IH, we hypothesize a relatively greater expression of PLK 1 in the hyperplastic intima of the injured vessels compared to the control vessels. This suggests that PLK 1 is involved in medial smooth muscle cell mitosis in the hyperplastic intima of injured vessels. In such a situation, the inhibitors of the PLK1 could be developed to inhibit the mitogenesis of VSMCs. This process would decrease the progression of the coronary IH, secondary to angioplasty or CABG, and hence may decrease the mortality and morbidity in CAD.

## Methods

### Porcine model

This experimental protocol has obtained formal approval from Creighton University Institutional Animal Care and Use Committee. The animals were housed in the Animal Resource Facility of Creighton University, Omaha, NE and cared for as per National Institute of Health standards. Yucatan™ miniature and microswine, weighing 20–45 kg, were purchased from Sinclair Bio-resources (Windham, MA). The swine were fed a special high cholesterol diet. The high cholesterol diet (HC) consists of 37.2% corn (8.5% protein), 23.5% soybean meal (44% protein), 20% chocolate mix, 5% alfalfa, 4% cholesterol, 4% peanut oil, 1.5% sodium cholate, and 1% lard; with 52.8% of the kilocalories from carbohydrates and 23.1% of the kilocalories from fat. After 16 weeks of the high cholesterol diet the animals were subjected to percutaneous transluminal balloon angioplasty (PTCA) in left anterior descending artery (LAD) or left circumflex artery (LCX) [32]. The control group of Yucatan™ and domestic swine were fed the normal diet but were excluded from undergoing PTCA.

Domestic swine were obtained from the UNL Swine Research Unit (Mead, NE). The domestic swine were fed a normal diet (Teklad Miniswine diet 8753, Harlan Laboratories) composed of wheat middlings, ground corn, soybean hulls, dehulled soybean meal, dehydrated alfalfa meal, dicalcium phosphate, and soybean oil. Within the diet, 57% of the kilocalories were from carbohydrates, 28% of the kilocalories from protein, and 15% of the kilocalories from fat. After one week of acclimatization, the animals were subjected to off-pump CABG; the right SEV was used as a vein graft.

### Anesthesia, monitoring, and operative procedures

Animals were sedated with a telazol and xylazine mixture prior to endotracheal intubation and general anesthesia was maintained with isoflurane. Analgesia was provided with local bupivacaine and IM buprenorphine. Animals had continuous EKG, rectal temperature, pulse oximetry, intra-arterial blood pressure and arterial blood gases monitoring in addition of the activated coagulation time monitoring during CABG. Animals were administered with prophylactic antibiotic cefazolin and prophylactic antiarrhythmic amiodarone and prophylactic anticoagulant heparin prior to the procedure. The defibrillator electrodes were attached preoperatively for defibrillation when needed.

### PTCA procedure

Transfemoral approach was adopted after femoral artery cut down. 6F arterial sheath was inserted and a 6F-guiding catheter was advanced to the left coronary artery on a guide wire under fluoroscopic guidance. Coronary angiography was done using nonionic contrast media. LCX or LAD balloon angioplasty were done followed by OCT (optical coherence tomography) to verify the placement and to get a baseline measurement of the surface are of both the LAD and LCX.

### CABG procedure

Off-pump CABG method was used. The SEV was harvested using the no touch technique through a longitudinal incision parallel to the mammary line. The chest was then opened through a sternotomy incision using the electric sternotomy saw. The heart was then exposed after incising the pericardium. The ascending aorta was exposed and prepared for the anastomosis using an aortic side clamp and a circular opening was created using a suitable aortic punch. The proximal part of the anastomosis between the proximal ascending aorta and the distal end of the SEV was completed. A cardiac surface stabilizer device was used to stabilize the area of the intended anastomosis with the LAD. The LAD was then dissected and incised; a temporary coronary shunt was used to maintain the coronary blood flow during the anastomosis. The distal part of the anastomosis between the LAD and the proximal end of the SEV was then completed. The temporary coronary shunt was removed before putting the last stitch. The blood flow in the bypass graft was verified before ligating the LAD proximal to the bypass to allow exclusive blood flow in the vein graft. The sternotomy was then closed using stainless wire and the soft tissues and skin were approximated and the animal was allowed to recover under thermal blanket and close monitoring during the postoperative recovery.

### Tissue harvest and processing

Heart was removed and placed in DMEM from both the swine models post PTCA and CABG. Coronary vessels (LAD, LCX, RCA), SEV-graft (right SEV) and left SEV were dissected, removed and fixed in 10% formalin for 24 hours at room temperature. Tissues were embedded in paraffin and thin sections (5μm) were obtained using microtome (Leica, Germany). Sections were stained with hematoxylin and eosin (H&E) for histomorphometric studies [33]. The non-injured vessels from swine with normal diet were used as controls.

### Immunofluorescence

Paraffin embedded samples, after deparaffinization and rehydration, were treated by steam heating for antigen retrieval (20–30 min) using DAKO antigen retrieval solution (DAKO, Carpenteria, CA). Slides were washed using Tris buffered saline and tween (TBST) twice 5 min each. Sections were incubated for 2 hr in block/permeabilizing solutions containing TBS, 0.25% Triton X-100, and 5% (v/v) goat serum at room temperature. The slides were subsequently incubated with primary antibody solutions including mouse polo like kinase1 (PLK1) (Abcam, ab17056), rabbit PLK1 phosphorylated at Thr210 (pPLK1) (Abcam, ab155095), mouse/rabbit non-muscle myosin heavy chain B (SMemb) (Abcam, ab684/ab24761), mouse smooth muscle alpha actin (α-SMA) (Abcam, ab7817), rabbit histone H3 phosphorylated at Ser-10 (pHis H3) (Abcam, ab32107), rabbit interferon gamma (IFN-γ) (Abcam, ab9657) and rabbit STAT3 phosphorylated at Tyr-705 (pSTAT3) (Bioss, bs-1658R) at 4°C overnight. On the following day, slides were rinsed in TBST three times for 5 minutes each, and the secondary antibody was incubated on the slides for 2 hours at room temp. In case of polyclonal primary antibodies, we used Alexa Fluor® 594-conjugated AffiniPure Goat Anti-Rabbit or Goat Anti-Mouse; Alexa Fluor 488-conjugated AffiniPure Goat Anti-mouse or Goat Anti-Rabbit secondary antibody, and for the monoclonal primary antibodies, we used Alexa Fluor 488 Goat Anti Mouse IgG2b secondary antibody, 1:200 (Jackson ImmunoResearch, Westgrove, PA). Negative controls with complete omission of primary antibody were run in parallel with normal host IgG. Sections were washed with TBST three times for 5 min. Nuclei were counterstained with 4’, 6- diamidino-2-phenylindole (DAPI) using Vector laboratories DAPI mounting medium. Tissue sections were viewed with an Olympus BX-51 epi-fluorescent microscope and images were photographed with an Olympus DP71 camera.

### Image analysis and fluorescence quantification

Numbers of immunopositive cells were counted and mean fluorescence intensity (MFI) was quantified using ImageJ software for each marker analyzed. Briefly, for each marker and in each independent experiment, 3 regions of interest (ROI) within the whole neointima were randomly chosen. The total numbers of cells showing immunopositivity for each marker and the MFI per cell in each of these ROI were analyzed. The ImageJ software quantified the protein expression in mean intensity value for each cell. The resulting values were then averaged and statistically analyzed.

### Statistical Analysis

In order to determine the statistical significance, data was analyzed using GraphPad Prism 5.0 biochemical statistical package (GraphPad Software, Inc., San Diego, CA). Values of all measurements are expressed as mean ± SEM of at least 3 independent experiments from each of the animals in each experimental group (2 for CABG; 5 for PTCA). Statistical analysis was performed using Student’s t-test to analyze statistically significant differences between the two groups. Differences at p<0.05 were considered significant.

## Results

### Vessel damage and development of intimal hyperplasia post PTCA and CABG

**Fig 1:**
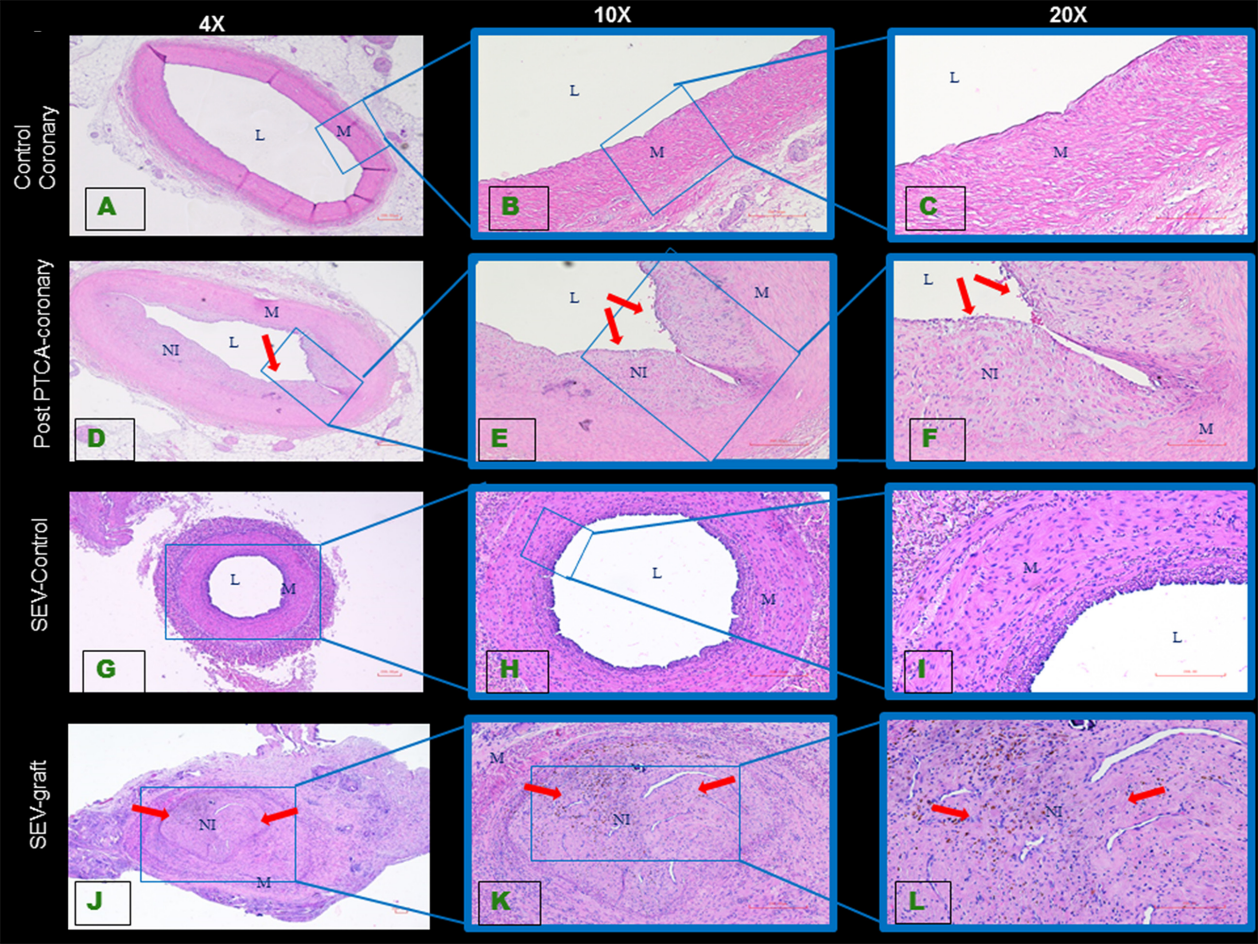
Histology of swine coronary artery and SEV tissue sections. The H&E staining of uninjured coronary artery (A, B, C) shows normal histology, whereas 16 weeks after balloon angioplasty neointimal hyperplasia (arrows) is noted in post PTCA-coronary arteries (D, E, F). H&E staining of the uninjured SEV showed normal histology (G, H, I). SEV that was used as a graft (J, K, L) developed neointimal hyperplasia (arrows). All images were taken in 4x, 10x and 20x objective. This is a representative of tissue histology in 2–5 individual swine in each experimental group. L: lumen, M: medial layer, NI: neointima.

Balloon injury resulted in medial rupture in all PTCA. Significant neointima proliferation was observed both in left anterior descending and left circumflex arteries following PTCA. Significant restenosis was observed in post PTCA-coronaries compared to RCA (Fig 1A–1F).

CAGB procedure exposes the SEV lumen to high pressure resulting in endothelial damage and dysfunction accompanied by medial SMC migration and proliferation. Significant IH of the SEV graft was observed compared to the left SEV that was not used as a graft (Fig 1G–1L).

### Vascular injury and PLK1 expression

**Fig 2:**
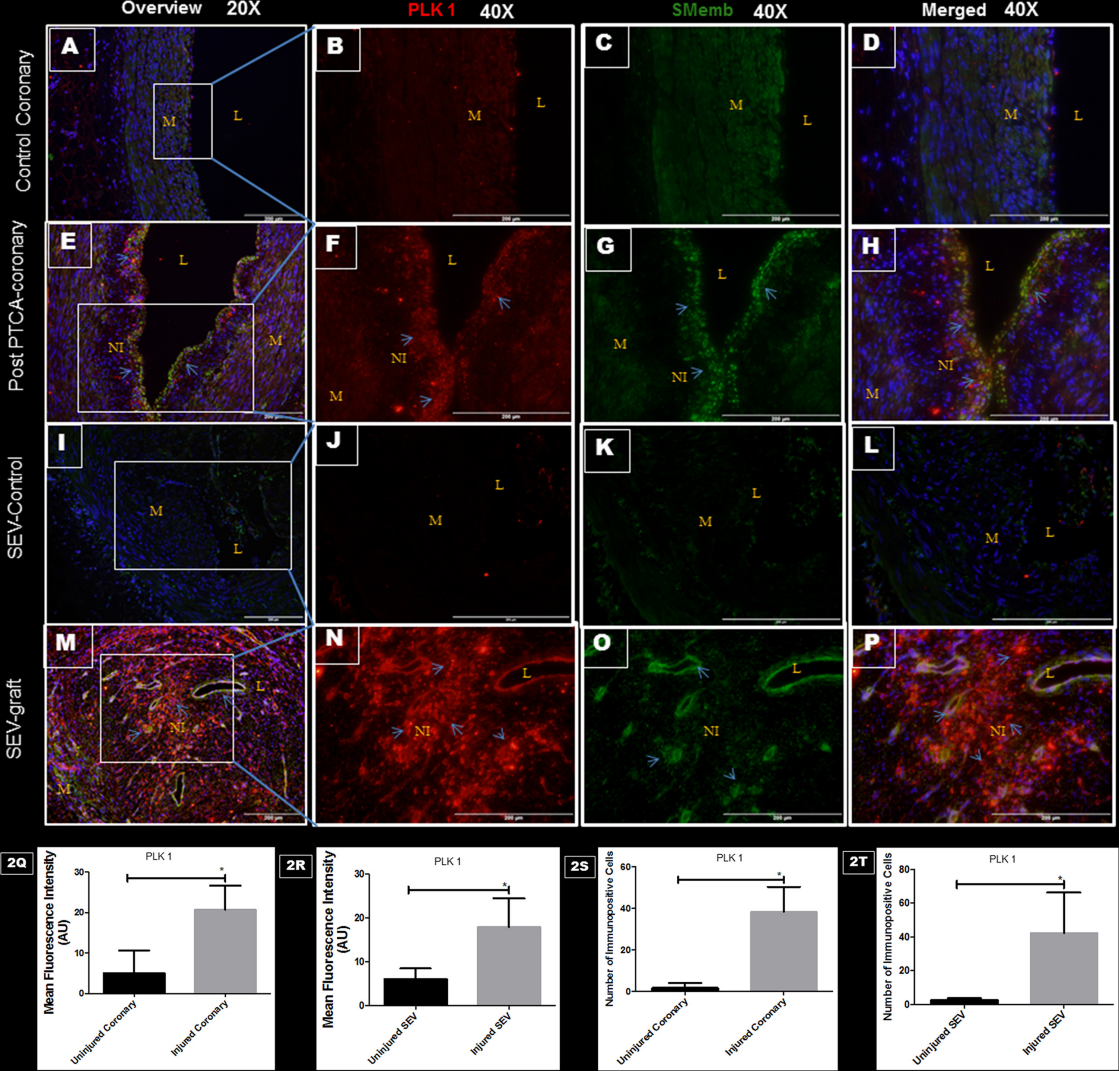
Double immunofluorescence showing the expression of PLK1 and SMemb in post PTCA- coronary arteries, SEV-grafts, and their uninjured counterparts in porcine model. There is less immunopositivity to PLK1 (red) and SMemb (green) expression in uninjured coronary artery at low (20x) (A) and high (40x) magnification (B,C,D). The immunostaining of PLK1 (red) and SMemb (green) is noted in restenotic post PTCA- coronary arteries (arrows) at low (20x) (E) and high (40x) magnification (F, G, H). Similarly, less immunostaining of PLK1 (red) and SMemb (green) expression is noted in the uninjured SEV at low (20x) (I) and high (40x) magnification (J, K, L) compared to SEV post CABG, where many cells showed immunopositivity to PLK1 (red) and SMemb (green) at low (20x) (M) and high (40x) (N,O,P) magnification. This is a representative of tissue histology in 2–5 individual swine in each experimental group. MFI measured in arbitrary units (AU) in post-PTCA coronary arteries (Q) and SEV-grafts (R) compared to their uninjured counterparts. Graphs showing number of PLK1- positive (+) cells in the neointima of the post-PTCA restenotic coronary artery (S) and SEV-grafts (T) compared to their uninjured counterparts. There was significantly increased immunopositivity to SMemb and PLK1 in the neointima of the post-PTCA restenotic coronary artery (arrows) compared to uninjured coronary artery. SEV-graft had significantly increased immunopositivity to SMemb and PLK1 in the neointima compared to uninjured SEV. Data are shown as mean ± SEM of values of at least three measurements in 2–5 individual swine in each experimental group. *P < 0.05. L: lumen, M: medial layer, NI: neointima.

Arteries injured by balloon angioplasty and SEV-graft was examined for PLK1 and SMemb expression by immunofluorescence. In order to be able to examine the co-distribution of both PLK1 and SMemb in the tissue, a double immunofluorescence procedure was used. Significantly increased expression of PLK1 and SMemb was noted in the post PTCA-coronaries and SEV-grafts compared to their respective uninjured counterparts (Fig 2). Few SMCs in the neointima expressed both PLK1 and SMemb. These results suggest a strong association between post-therapeutic interventional vascular injury and PLK1 expression.

### Vascular injury and pPLK1 expression

**Fig 3:**
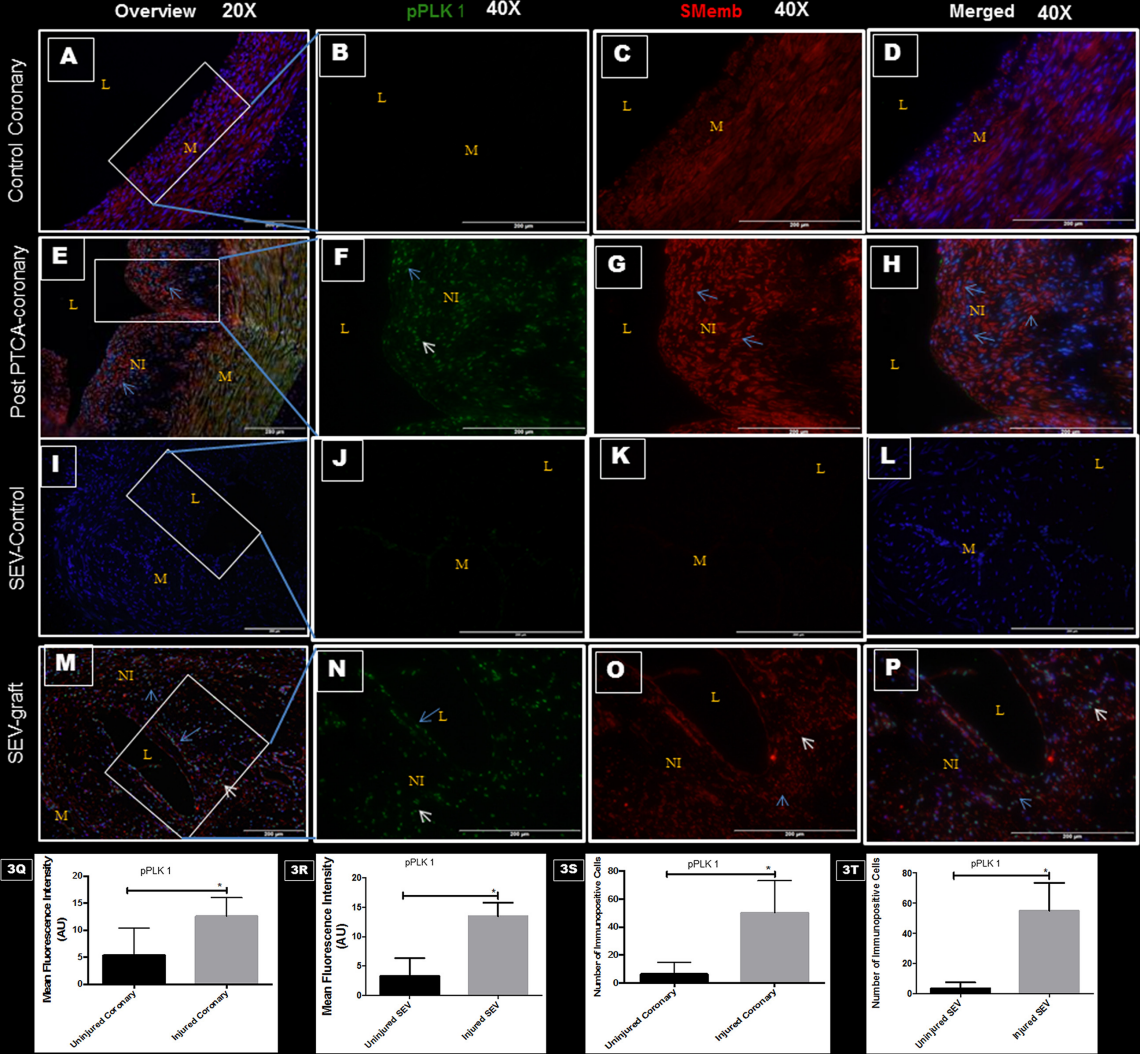
Double immunofluorescence showing the expression of pPLK1 and SMemb in post- PTCA coronary arteries, SEV-grafts, and their uninjured counterparts in swine. There is less immunopositivity to pPLK1 (green) and SMemb (red) expression in uninjured coronary artery at low (20x) (A) and high (40x) magnification (B,C,D). The immunostaining of pPLK1 (green) and SMemb (red) is noted in restenotic post PTCA- coronary arteries (arrows) at low (20x) (E) and high (40x) magnification (F, G, H). Similarly, less immunostaining of pPLK1 (green) and SMemb (red) expression is noted in the uninjured SEV shown at low (20x) (I) and high (40x) magnification (J, K, L) compared to SEV post CABG, where many cells showed immunopositivity to pPLK1 (green) and SMemb (red) at low (20x) (M) and high (40x) (N, O, P) magnification. This is a representative of tissue histology in 2–5 individual swine in each experimental group. MFI measured in arbitrary units (AU) in post-PTCA coronary arteries (Q) and SEV-grafts (R) compared to their uninjured counterparts. Graphs showing number of pPLK1- positive (+) cells in the neointima of the post-PTCA restenotic coronary artery (S) and SEV-grafts (T) compared to their uninjured counterparts. There was significantly increased immunopositivity to SMemb and pPLK1 in the restenotic post-PTCA coronary arteries compared to uninjured coronary arteries. SEV-graft had significantly increased immunopositivity to SMemb and pPLK1 in the neointima compared to uninjured SEV. Data are shown as mean ± SEM of values of at least three measurements in 2–5 individual swine in each experimental group. *P < 0.05. L: lumen, M: medial layer, NI: neointima.

Post PTCA-arteries and SEV used as a coronary bypass graft were examined for pPLK1 and SMemb expression by immunofluorescence. Phosphorylation of PLK1 at Thr-210 by other kinases is required for its catalytic activity. Restenotic coronary artery post PTCA showed significant expression of pPLK1, which was limited to the neointima; however, SMemb was expressed in the neointima and also in the medial layer, compared to the uninjured RCA. Few cells in the neointima expressed both pPLK1 and SMemb (Fig 3A–3H, 3Q and 3S). Moreover, significantly increased expression of SMemb and pPLK1 were noted in the neointima of the SEV-graft compared to uninjured left SEV (Fig 3I–3P, 3R and 3T). In the SEV-graft, a group of cells in the neointima expressed either SMemb and pPLK1 or a combination of both. These results suggest a strong association between post-therapeutic interventional vascular injury and pPLK1 expression.

### Vascular injury, pHistone-H3 and SMemb

**Fig 4:**
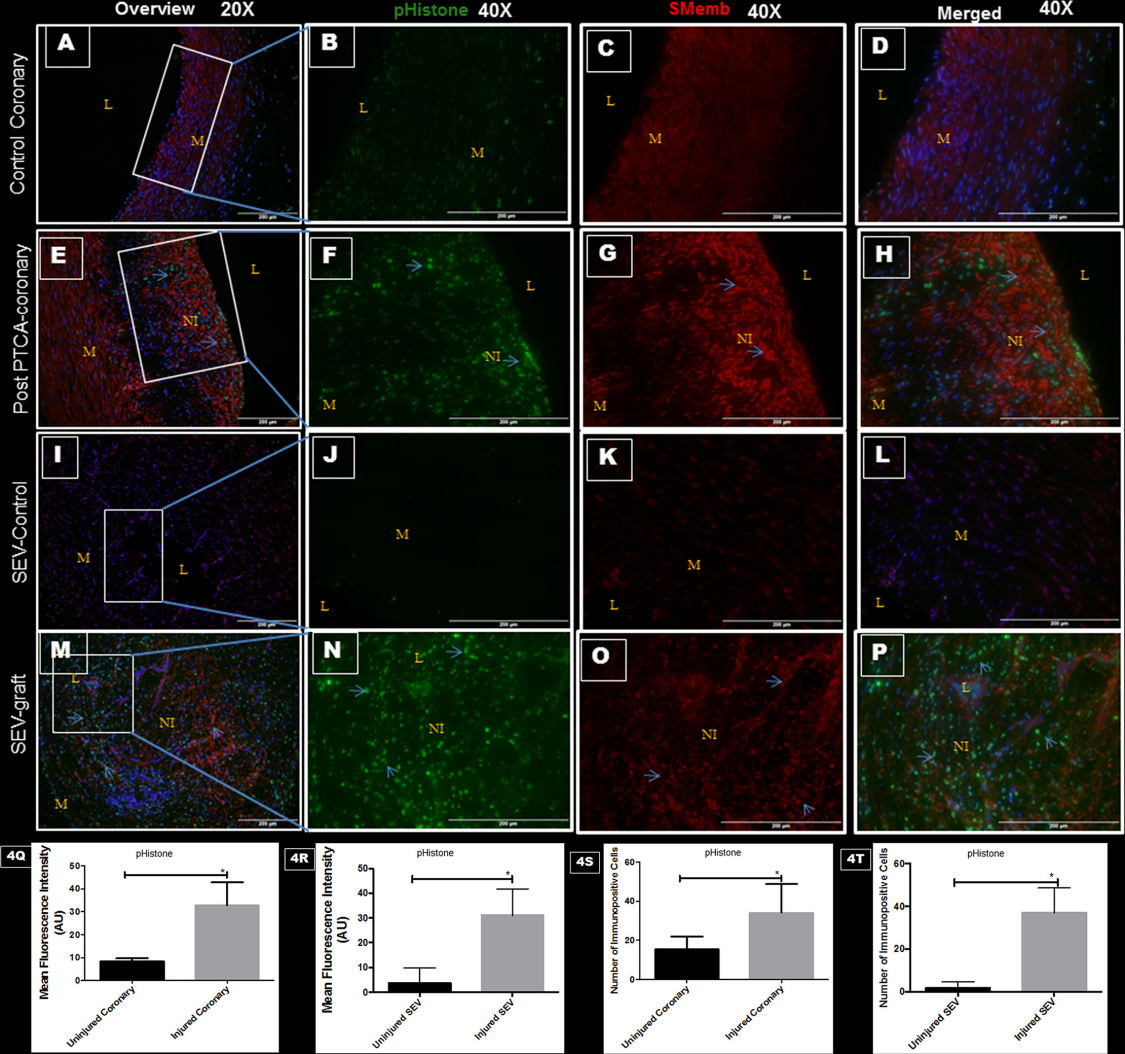
Double immunofluorescence showing expression of pHistone H3 at Ser-10 and SMemb in thin sections of uninjured swine coronaries, post-PTCA coronary arteries, uninjured swine SEV and SEV-graft. There is less immunopositivity to pHistone (green) and SMemb (red) expression in uninjured coronary artery shown at low (20x) (A) and high (40x) magnification (B, C, D). The immunostaining of pHistone (green) and SMemb (red) is noted in restenotic post PTCA- coronary arteries (arrows) at low (20x) (E) and high (40x) magnification (F, G, H). Similarly, less immunostaining of pHistone (green) and SMemb (red) expression is noted in the uninjured SEV shown at low (20x) (I) and high (40x) magnification (J, K, L) compared to SEV post CABG, where many cells showed immunopositivity to pHistone (green) and SMemb (red) at low (20x) (M) and high (40x) (N,O,P) magnification. This is a representative of tissue histology in 2–5 individual swine in each experimental group. MFI measured in arbitrary units (AU) in post-PTCA coronary arteries (Q) and SEV-grafts (R) compared to their uninjured counterparts. Graphs showing number of pHistone- positive (+) cells in the neointima of the post-PTCA restenotic coronary artery (S) and SEV-grafts (T) compared to their uninjured counterparts. Strong immunopositivity to pHistone H3 was found in the neointima of the post PTCA-coronary arteries and in SEV-graft. Many cells in the neointima of the injured vessels were immunopositive for both, pHistone H3 and SMemb, especially around the pseudo lumen in SEV-graft. Data are shown as mean ± SEM of values of at least three measurements in each group in 2–5 individual swine in each experimental group. *P < 0.05. L: lumen, M: medial layer, NI: neointima.

Immunoreactivity towards phospho-histone H3 (pHistone) at Ser-10 was done to examine proliferating cells in the neointimal area. Phosphorylation at a highly conserved serine residue (Ser-10) in the histone H3 tail is considered to be a crucial event for the onset of mitosis. Significantly increased immunopositivity towards pHistone and SMemb were noted in the same tissue sections of post PTCA coronaries and SEV-grafts compared to their respective uninjured counterparts (Fig 4). Immunopositivityy towards pHistone was noted only in the cells at the neointima in both post PTCA-coronaries and SEV-graft. Many cells in the neointima of the injured vessels were immunopositive for both pHistone at Ser-10 and SMemb.

### Vascular injury, SMemb and smooth muscle α-actin

**Fig 5:**
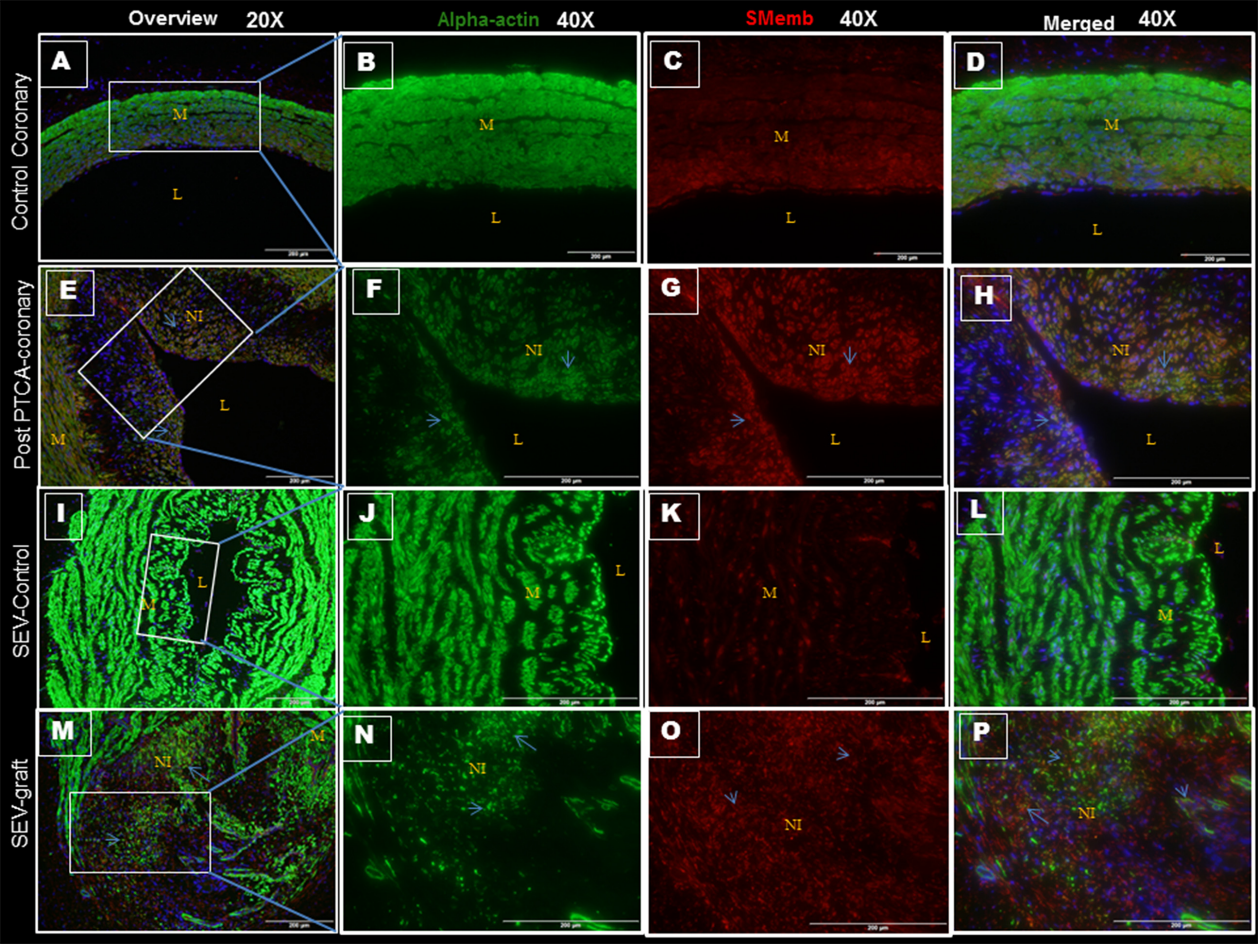
Double immunofluorescence showing expression of smooth muscle α-actin (α-SMC) and SMemb in thin sections of uninjured coronaries, post PTCA-coronary arteries, uninjured SEV and SEV-grafts in swine. Expression of α-SMC (green) and SMemb (red) are noted in uninjured coronary artery at low (20x) (A) and high (40x) magnification (B, C, D). Less expression of α-SMC (green) and increased expression of SMemb (red) are found in the hyperplastic region post PTCA- coronary arteries (arrows) at low (20x) (E) and high (40x) magnification (F, G, H). Similarly, immunopositivity to α-SMC (green) and SMemb (red) are observed in the uninjured SEV at low (20x) (I) and high (40x) magnification (J, K, L) compared to SEV post CABG, where few cells are immunopositive to α-SMC (green) and many cells showed immunopositivity to SMemb (red) at low (20x) (M) and high (40x) (N, O, P) magnification. Increased density of SMemb-immunopositive cells are found at the neointima in post PTCA- coronary arteries and SEV-grafts compared to that of normal uninjured coronary artery and vein, respectively. Strong expression of α-SMA is noted in all the vessels, injured or uninjured. This is a representative of tissue histology in 2–5 individual swine in each experimental group. L: lumen, M: medial layer, NI: neointima.

Immunopositivity towards SMemb in both post PTCA-coronaries and SEV-graft establishes the presence of synthetic SMCs with increased rate of proliferation (Fig 5). High expression of smooth muscle α-actin (α-SMA) was found in all the vessels. Expression of α-SMA and SMemb in these lesions confirmed that vascular SMCs were the main cellular component of neointimal proliferative lesions. There is an increased expression of SMemb at the neointima in post PTCA-coronaries and SEV-grafts compared to that of their uninjured counterparts. In PTCA-coronaries, SMemb was mostly expressed in the neointima; some of these SMCs also expressed α-SMA. However, in the SEV-grafts, two distinct sets of SMCs were observed, one expressed SMemb and the other expressed α-SMA. A few SMCs around the pseudo-lumen showed co-localization of both the markers.

### Vascular injury, IFNγ and SMemb

**Fig 6:**
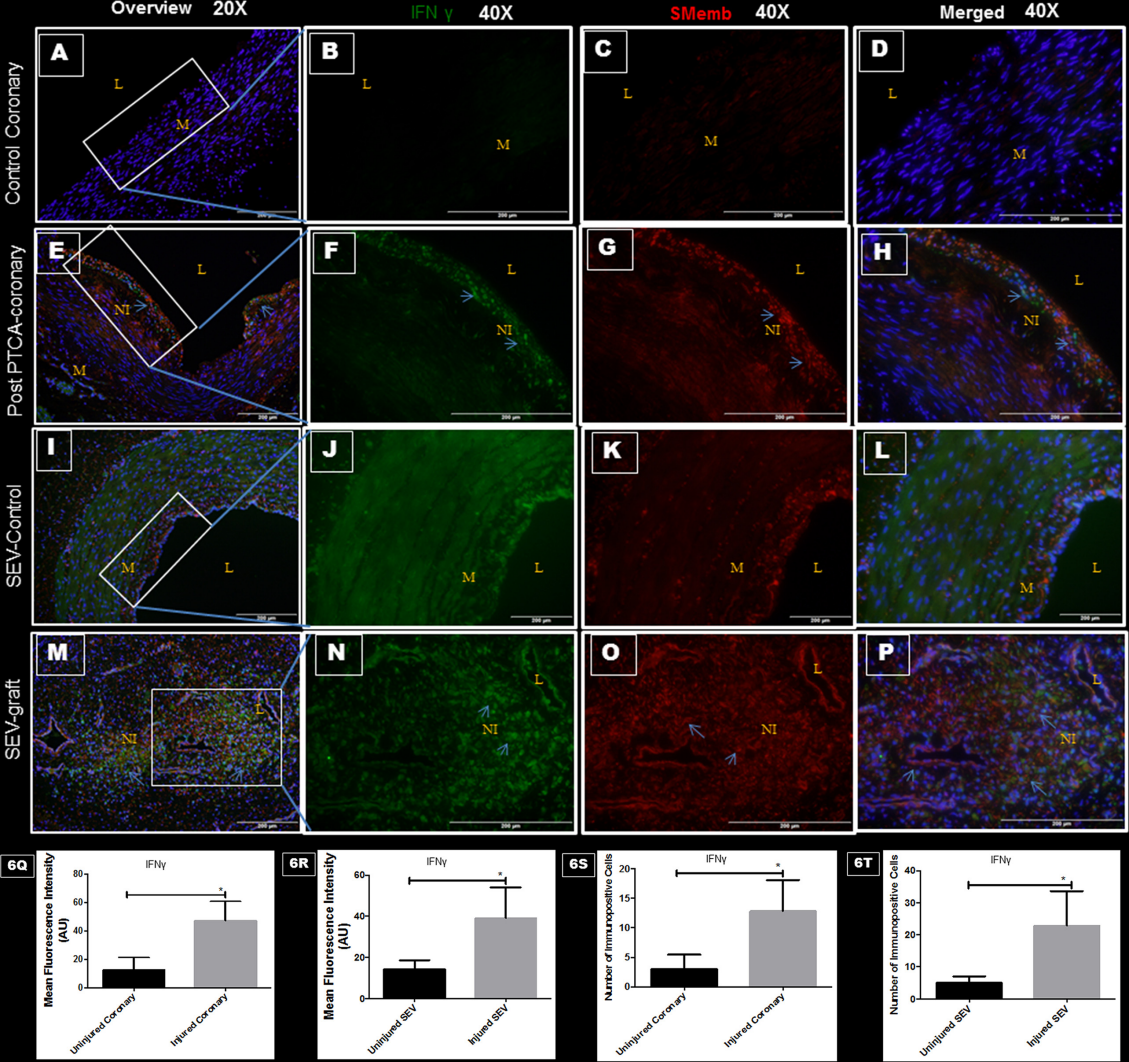
Double immunofluorescence showing expression of IFNγ (green) and SMemb (red) in thin sections of uninjured coronaries, post PTCA-coronary arteries, uninjured SEV and SEV-graft. Less immunopositivity to IFNγ (green) and SMemb (red) are noted in uninjured coronary artery at low (20x) (A) and high (40x) magnification (B, C, D). Strong expression of IFNγ (green) and SMemb (red) are found in the hyperplastic region post PTCA- coronary arteries (arrows) at low (20x) (E) and high (40x) magnification (F, G, H). Similarly, less immunostaining of IFNγ (green) and SMemb (red) expression is noted in the uninjured SEV at low (20x) (I) and high (40x) magnification (J, K, L) compared to SEV post CABG, where many cells showed immunopositivity to IFNγ (green) and SMemb (red) at low (20x) (M) and high (40x) (N,O,P) magnification. This is a representative of tissue histology in 2–5 individual swine in each experimental group. MFI measured in arbitrary units (AU) in post-PTCA coronary arteries (Q) and SEV-grafts (R) compared to their uninjured counterparts. Graphs showing number of IFNγ - positive (+) cells in the neointima of the post-PTCA restenotic coronary artery (S) and SEV-grafts (T) compared to their uninjured counterparts. Many cells in the neointima of the injured vessels were immunopositive for both, IFNγ and SMemb, especially around the pseudo lumen in SEV-graft. Data are shown as mean ± SEM of values of at least three measurements in each group in 2–5 individual swine in each experimental group. *P < 0.05. L: lumen, M: medial layer, NI: neointima.

A double immunofluorescence procedure was used to examine the immunoreactivity towards IFNγ and SMemb in the same tissue. Significantly increased expression of IFNγ and SMemb was noted in post PTCA-coronaries and SEV-grafts compared to the uninjured counterparts (Fig 6). The expression of IFNγ was limited to the neointima of the injured vessel. Most of the SMCs in the neointima of post PTCA-coronaries and SEV-grafts co-expressed IFNγ and SMemb.

### Vascular injury, pStat-3 and PLK1

**Fig 7:**
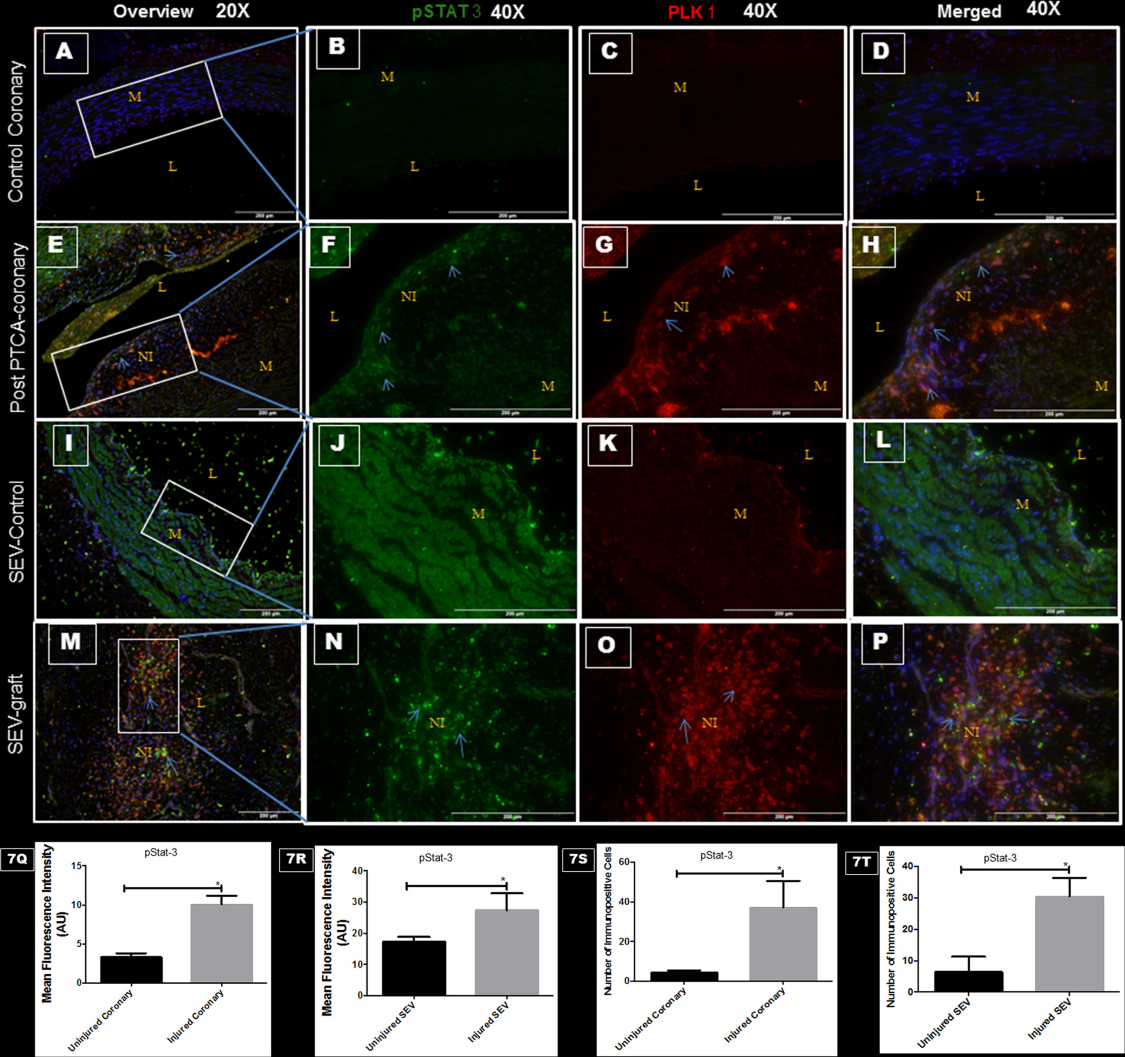
Double immunofluorescence showing expression of pStat-3 (green) and PLK1 (red) in thin sections of uninjured coronaries, post PTCA-coronary arteries, uninjured SEV and SEV-graft in swine. Less immunopositivity to pStat-3 (green) and PLK1 (red) are noted in uninjured coronary artery at low (20x) (A) and high (40x) magnification (B, C, D). Strong expression of pStat-3 (green) and PLK1 (red) are found in the hyperplastic region post PTCA- coronary arteries (arrows) at low (20x) (E) and high (40x) magnification (F, G, H). Similarly, less immunostaining of pStat-3 (green) and PLK1 (red) expression is noted in the uninjured SEV at low (20x) (I) and high (40x) magnification (J, K, L) compared to SEV post CABG, where many cells showed immunopositivity to pStat-3 (green) and PLK1 (red) at low (20x) (M) and high (40x) (N,O,P) magnification. This is a representative of tissue histology in 2–5 individual swine in each experimental group. MFI measured in arbitrary units (AU) in post-PTCA coronary arteries (Q) and SEV-grafts (R) compared to their uninjured counterparts. Graphs showing number of pStat-3—positive (+) cells in the neointima of the post-PTCA restenotic coronary artery (S) and SEV-grafts (T) compared to their uninjured counterparts. Increased immunopositivity towards pStat-3 and PLK1 are found in the neointimal region of the injured vessels compared to the uninjured vessels. This is a representative of tissue histology in 2–5 individual swine in each experimental group. L: lumen, M: medial layer, NI: neointima.

Significantly increased expression of pStat-3 and PLK1 was noted in post PTCA-coronary arteries and SEV-grafts compared to the uninjured counterparts (Fig 7). Many SMCs in the neointima of the injured vessels showed immunopositivity towards both pStat-3 and PLK1.

## Discussion

Restenosis after percutaneous coronary intervention, and vein graft failure post CABG, are chronic pathological conditions that proceed to obstructive vascular lesions with time. Mitogenic stimuli released at the site of the injury elicit different signaling pathways, but all finally converge at the cell cycle of the proliferating medial smooth muscle cells. The process of DNA doubling (S phase) and distribution (mitosis) are separated by G1 and G2 phases, respectively. In the G1- and G2-phases of cell cycle, the mitogenic and anti-mitogenic signals are combined and the cells exit, pause or continue through the cell cycle. Beyond these two R-points (G1 and G2), cells are committed to further progress through the cell cycle, independent of extracellular stimuli [34]. Reports have shown that at the G1 R-point, p27KIP1, Rb, PTEN proteins are down regulated in mitogen stimulated vascular SMCs increasing the transcription and stabilization of cyclin D, which critically contributes to the development of IH [35]. However, the second restriction site in the cell cycle, G2 R-point, may prevent proliferation of the cells, which have lost proper G1/S control. Plk1, activated during G2-M phase, activates CDC25 and controls the activity of Cdk1/Cyclin B1 complex thus promoting the G2/M phase transition [36–41]. Injection of anti-PLK1 antibodies in HeLa cells mitotically arrested the cells to divide [42].

Extensive volume of literature cites the association of PLK1 with G2-M transition and mitosis in numerous cancer conditions [36–41]. Therefore, it is plausible that an association, between phenotypically altered SMCs and PLK1 also exists in hyperplastic cells found in non-neoplastic pathologies such as IH. In this study we have demonstrated for the first time the increased expression and activity of PLK1 in SMCs present in the hyperplastic zone in vessels exposed to post- therapeutic intervention.

Vascular injury caused by PTCA and CABG leads to inflammatory cell infiltration; these cells release IFNγ, which plays an important role in initiating IH. IFNγ stimulates the transcriptional activity of pro-proliferative genes through Stat-3 and causes SMC phenotype switching and proliferation leading to IH. PLK1 is one of the target genes of stat-3 [21, 23–25]. Phenotypically transformed SMCs proliferate at a higher rate, express distinct synthetic markers including SMemb, and possess decreased contractile proteins; when compared to the quiescent smooth muscle cells found in the tunica media of normal vessels [22].

Neointimal thickening was observed in arteries post-PTCA and SEV-grafts compared to their uninjured counterparts. In this study we have documented the increase in the number of PLK1- positive (+) cells in the neointima of restenotic coronary arteries, post balloon angioplasty and post CABG SEV grafts, compared to the respective uninjured counterparts. Many of the SMCs in the neointimal region, which were positive for PLK1, were also found to be immunopositive for SMC synthetic marker SMemb. PLK1 undergoes phosphorylation at Thr-210 and becomes catalytically active [43]. We showed the co-distribution of both pPLK1 and SMemb in the hyperplastic region of coronary arteries and SEVs post intervention or surgery. The neointimal-SMCs expressing immunopositivity to pPLK1 is significantly high to that of the uninjured vessels. Increased expression of SMemb was not only noted in the neointima but also in the medial layer of the occluded vessel compared to the uninjured and un-occluded ones. These results indicate that the phenotypically modified SMCs in the neointima were over expressing PLK1. In the post CABG SEV graft sections we found a group of cells in the hyperplastic zone that showed immunopositivity for SMemb, PLK1 and pPLK1. These results reveal a strong association between vascular injury and pro-proliferative mitotic kinase PLK1 expression and activity in post PTCA coronary arteries and SEV-grafts. The potential ability of SMCs to replicate under mitogenic stimuli could occur through the loss of negative growth regulators and/or to the overexpression of pro-proliferative protein.

We also observed immunopositivity in the neointimal area towards pHistone H3 at Ser-10 that correlates with the cells undergoing mitosis [44, 45]. Significant increase in the number of the pHistone-positive SMCs was noted in balloon-injured coronaries and SEV grafts compared to that of their uninjured vessels. The expression of pHistone was limited only to the neointima and many of these cells also co-expressed SMC-synthetic marker SMemb and pHistone. This observation may be due to the high proliferative nature of the synthetic SMCs. As mentioned above many of these highly proliferative cells in the neointima were also positive for PLK1 and pPLK1. To the best of our knowledge, ours shall be the first study, which demonstrates increased expression and activity of PLK1 in the neointimal region post vascular injury along with the expression of SMC-synthetic marker SMemb and mitotic marker pHistone H3. Next, we wanted to know if these cells express the contractile SMC marker, α-SMA [22]. Strong α-SMA was observed in all the vessels, injured or uninjured, but SMemb was only expressed in the neointima of the injured vessels. In post PTCA-coronaries, many cells in the neointima expressed both α-SMA and SMemb, however, in the SEV grafts two distinct sets of cells expressed α-SMA and SMemb. This could be due to the higher rate of phenotypic transformation of medial VSMCs in post CABG venous grafts, compared to that of the post PTCA-coronary arteries. Venous grafts are more sensitive to shear stress than coronary arteries and this would explain the pronounced IH in the venous grafts compared to the post PTCA- coronary arteries. Significantly increased expression of IFN-γ and active transcription factor pStat-3 were noted in vessels post PTCA and CABG. Increased number of cells expressing IFN-γ and pStat-3 were observed in the neointima of both, post PTCA-coronaries and SEV-grafts compared to the uninjured vessels. IFN-γ was mostly co-expressed with SMemb in the neointima of the vessels. This could explain that synthetic SMCs are one of the cellular sources of IFN-γ. Many phosphorylated Stat-3 positive cells were also immunopositive for PLK1. Studies have shown that IFN-γ can induce phosphorylation of stat-3 which in-turn can transcribe PLK1 gene. In this study we found more cells in the neointima expressing immunopositivity towards SMemb, pHistone H3, PLK1, pPLK1, IFN-γ and pStat-3 in the post PTCA- coronary arteries and SEV-grafts compared to their respective uninjured counterparts. Therefore, an increased MFI of each immunofluorescent stain within the injured vessels is due to a greater number of immunopositive cells in the neointima. This suggests that each of these markers/proteins may have played a critical role in the formation of the intimal hyperplasia post vascular injury. This study should pave the way for further in-vitro studies to understand the relationship among these proteins in regard to IH. Also, future studies are warranted to further understand the role of mitotic kinase PLK1 in the pathogenesis and prognosis of IH in coronary arteries following intervention and in vein graft disease.
